# Does the birth plan match what is relevant to women? Preferences of Spanish women when giving birth

**DOI:** 10.1186/s12905-023-02856-5

**Published:** 2024-01-15

**Authors:** Isabel Artieta-Pinedo, Carmen Paz-Pascual, Arturo Garcia-Alvarez, Paola Bully, Ema-Q Group, Isabel Artieta-Pinedo, Carmen Paz-Pascual, Sonia Alvarez, Pilar Amorrortu, Mónica Blas, Inés Cabeza, Itziar Estalella, Ana Cristina Fernández, Gloria Gutiérrez de Terán-Moreno, Kata Legarra, Gorane Lozano, Amaia Maquibar, David Moreno-López, Ma. Jesús Mulas, Covadonga Pérez, Angela Rodríguez, Mercedes Sáenz de Santamaría, Jesús Sánchez, Gema Villanueva, Maite Espinosa

**Affiliations:** 1 Primary Care Midwife OSI Barakaldo Sestao, Osakidetza, Barakaldo, Spain; 2 Biobizkaia Health Research Institute, Plaza de Cruces 1, 48903 Bizkaia, Barakaldo Spain; 3 Department of Nursing I, Faculty of Medicine and Nursing, University of the Basque Country (UPV/EHU), Basque, Bizkaia Spain; 4 Midwifery Training Unit of Basque Country, Bilbao, Spain; 5 Servicio Vasco de Salud-Osakidetza, Vitoria-Gasteiz, Alava Spain; 6 Methodological and Statistical Consulting, Sopuerta, Bizkaia Spain

**Keywords:** Shared decision-making, Birth preference, Birth plan, women’s views, Maternity care

## Abstract

**Background:**

To support women in making shared decisions, it is important to know what is relevant to them. The aim is to explore which of the options included in birth plans (BP) are of most interest to women, and which are more controversial. In addition, the possible association of this variability with personal characteristics.

**Methods:**

The data are part of a cross-sectional descriptive study, carried out in xxx, on the clinimetric characteristics of two instruments to measure women’s needs in labour and postpartum. Women were recruited consecutively by their midwives during pregnancy check-ups, receive a link to a digital questionnaire and were allowed to provide links to the questionnaires to other pregnant women. Women were asked to determine their level of agreement with statements about the birth environment, accompaniment, pain relief, medical intervention and neonatal care. The relationship between agreement with each statement, socio-demographic variables and fear of childbirth (W-DEQ-A) was analysed using a combination of descriptive statistics to analyse frequencies, and regression models to test the effect of socio-demographic variables and fear of childbirth on those items with the greatest variability.

**Results:**

Two hundred forty-seven women responded. More than 90% preferred a hospital delivery, with information about and control over medical intervention, accompanied by their partner and continuous skin-to-skin contact with the newborn. There are other questions to which women attach less importance or which show greater variability, related to more clinical aspects, like foetal monitoring, placenta delivery, or cord clamping… Various factors are related to this variability; parity, nationality, educational level, risk factor or fear of childbirth are the most important.

**Conclusions:**

Some items referring to the need for information and participation are practically unanimous among women, while other items on technical interventions generate greater variability. That should make us think about which ones require a decision after information and which ones should be included directly. The choice of more interventional deliveries is strongly associated with fear of childbirth.

**Supplementary Information:**

The online version contains supplementary material available at 10.1186/s12905-023-02856-5.

## Introduction

Involving people in decisions about their health and care improves their level of health and well-being, improves the quality of care, and ensures that people make informed use of available health resources [1]. In shared decision-making, clinicians and patients can analyse the available information and work together to reach a decision that takes into account the preferences and values of each patient [2].This increases satisfaction [3] and, in the case of childbirth, seems to reduce symptoms of perinatal depression, preterm births and low birth-weight newborns [2].

A birth plan is intended to be a tool for helping make shared decisions regarding the birth of the baby, as it is a written document in which the pregnant woman expresses her wishes and expectations for the moment of the delivery and postpartum [4, 5]. In 2008, the Ministry of Health, Social Policy and Equality of Spain drew up a Birth Plan document outlining the options that women can select throughout the process [6]; and most hospitals offer similar plans, with some differences in the amount of information that accompanies each option [7]. The model proposed by the Ministry of Health serves as a template for each hospital to develop its own birth plan document according to its available resources. Following this script, the woman reflects on and confirms in writing her preferences for childbirth. It is divided into 7 sections: 1) arrival at the hospital, which is a section that allows for the choice of a companion and their degree of participation in the process, as well as special needs due to capacity, culture or language, and the issue of physical space; 2) dilation period, which includes the choice of preferred place and position to facilitate delivery, pain management methods, use of support material and other care preferences, as well as information about possible interventions; 3) expulsion period, a section that includes questions about preferences regarding skin-to-skin contact, umbilical cord clamping and/or desire to donate blood from it; 4) delivery of the placenta, and whether this should happen spontaneously or be managed; 5) care and attention of the newborn, which includes the question of the mode of administration of vitamin K to the baby (oral or intramuscular) and responsibility for the care and hygiene of the baby; 6) postpartum period, which includes preferences regarding the method of breastfeeding and if support is desired in this matter, as well as aspects related to mother-child cohabitation and 7) instrumental delivery or caesarean section, where any preference is to be stated if labour has to be induced (a technical issue decided by professionals in public hospitals) [6].

Some studies conclude that the birth plan can be an effective tool for promoting a more natural and physiological birth process, better communication with professionals, greater control of the birth process itself, better obstetric and neonatal outcomes, and a greater degree of satisfaction [8, 9]. However, the possibility of choosing between a high number of options within the Birth Plan is not necessarily associated with greater satisfaction if a high percentage of what is requested is not subsequently carried out [10]. Indeed, a recent study carried out in our country showed that the birth plan was only complied with for the most part (≥75% of the indicated preferences) in 37% of cases [11], which could be related to lower satisfaction with the birth experience [12]. Furthermore, it is possible that having a high number of options increases the expectation of an ideal birth, which can lead to disappointment and leave the woman without resources in the face of unexpected events [12].

If the birth plan is intended to be a tool to help make shared decisions about the birth and the arrival of the newborn, its content must be neither too long nor too short, it must be achievable, it must encourage communication and, above all, it must be relevant to the woman. For this last requirement, the study of preferences is important. Some studies have assessed women’s preferences during childbirth [13, 14]; most have focused on the choice of delivery place [15–17], the type of delivery [18] or the type of analgesia to be used [19, 20]. There are other issues, some included in the birth plan and some not, that could have an impact on maternal well-being during the process, and require further study.

The objective of this study is: 1) to expand the information available on women’s preferences in aspects such as comfort, support and medical intervention during childbirth; how much importance they attach to these issues and how much consensus there is among women about their importance; 2) compare these preferences with what is offered in the Ministry’s Birth Plan to assess whether there is a correlation between what is asked and what is really relevant to them; 3) we also will focus on the questions that present the greatest variability, in order to analyze whether this variability is associated with certain sociodemographic characteristics or fear of childbirth.

## Methods

### Design and selection of participants

The data is part of a broader investigation that analyses the needs of women during pregnancy, childbirth and postpartum, and the resources they have available to them to adapt to the new situation. The study protocol and results have been published previously [21, 22]. It is a cross-sectional study, carried out in the xxx, which serves a population of just over 2 million inhabitants. Each of the six hospitals with maternity services coordinates with a set of 64 primary care centres for monitoring pregnancy, childbirth and postpartum. The annual number of births is approximately 14,000 [23].

In the context of the previous study [22] - based on the findings in a pilot test, the length and characteristics of the questionnaire, and the possible effect of other variables - it was considered that a sample size greater than 200 offered adequate statistical power. Thus, between March and October 2020, a consecutive sampling of pregnant women was carried out in 20 midwives’ offices until the number of 250 completed questionnaires was reached. This meant recruiting a few more women, due to the way of accessing the questionnaires, through a link that the midwife provided to the woman.

About 1000 pregnant women attended the midwives’ offices during the study period. The women who participated were recruited, in addition to consecutively by their midwives in a pregnancy check-up, through information provided by the women themselves (snowball sampling). They offered women who met the inclusion criteria to participate; if they accepted, they were provided with a link through their cell phones, which gave them access to the questionnaires in digital format. Only women under 18 years of age, or those who did not understand Spanish fluently enough to answer the questions, were excluded from the participation in the study. 15–20% of the population attended did not meet the criterion of sufficient linguistic competence to be able to perform the test, since the proportion of foreigners among the pregnant women is usually high. Women with high-risk pregnancies were not included either, since the recruitment was carried out in the midwives’ offices (in our health system, the midwife is in charge of attending non-pathological pregnancies, while high-risk pregnancies are attended by the obstetrician). A specific gestational age was not established because many decisions regarding childbirth are made even before pregnancy [16, 18].

When the woman followed the link, she received information about the characteristics of the study, and a request for informed consent that, once accepted, allowed access to the questionnaire. All responses were collected in an encrypted password-protected online database. The study was approved by the xxx Ethical Committee (PI2019110).

**Fig. 1:**
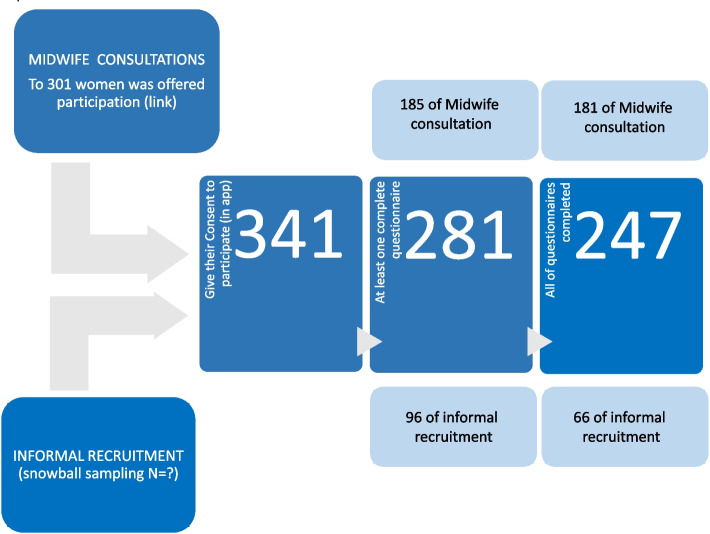
Flow-chart of the study

Three hundred forty-one women finally gave their consent to participate in the digital application and, of them, 247 women responded to the total number of questionnaires (See Fig. 1. Flowchart).

### Measurements

**Fig. 2:**
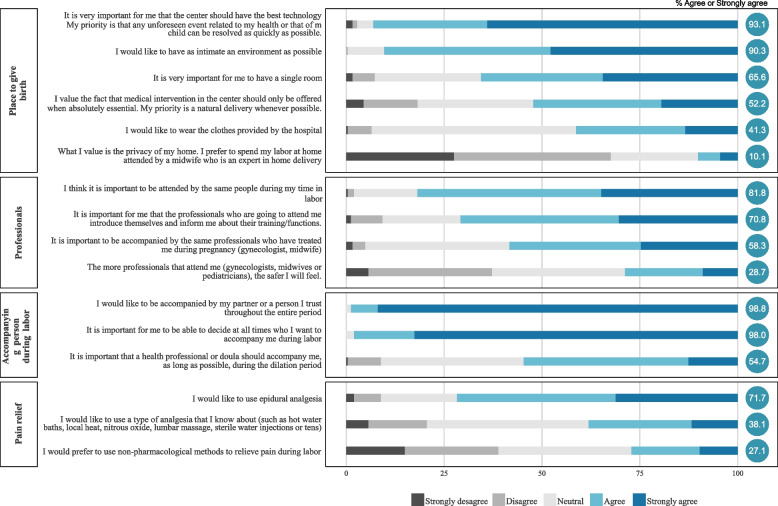
Percentage of women in each alternative for each option regarding the environment, accompanying person and pain relief during childbirth

**Fig. 3:**
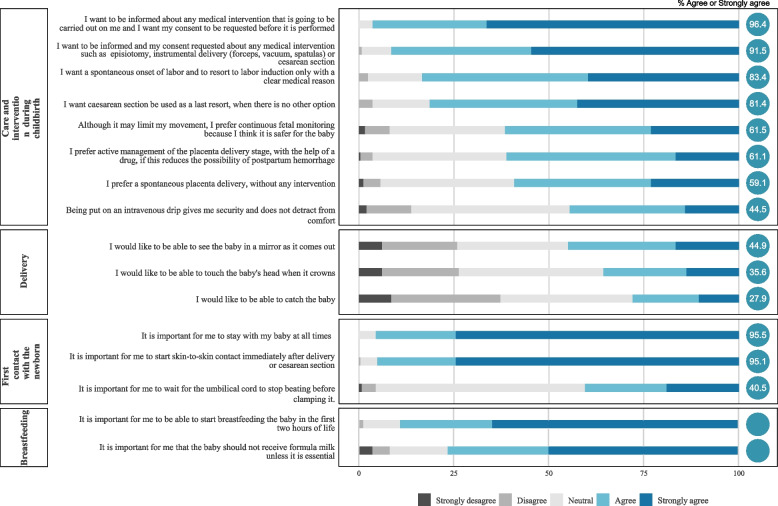
Percentage of women in each alternative for each of the statements about options during childbirth, immediate postpartum and newborn care

To study the preferences, a list of 32 frequent statements in the birth plan documents grouped into blocks was drawn up [6, 7, 13, 23]: desired place (6 questions); presence of professionals [3] and companions [4]; pain relief [3]; acceptance of medical intervention [8]; delivery period [3]; immediate care [3] and feeding of the newborn [2]. Statements were framed as “It is important for me...” and showed various possible options. Each woman responded according to her degree of agreement from: 1 (totally disagree) to 5 (totally agree). The blocks with the items in each of them are shown in Figs. 2 and 3. The questions are part of a larger questionnaire designed to detect the psychosocial needs of women during the perinatal period and which has been validated with 250 women, presenting good characteristics of reliability, validity and usability. The protocol and results have been published [21, 22]. The degree of agreement or disagreement with each of the expressed preferences was considered the outcome variable.

The possible effect of some explanatory variables of the variability in preferences was taken into account. Fear of childbirth, age, parity (none/one or more children) [24], nationality (Spanish/foreign) [25, 26], level of education (low/medium/high), paid work (yes/no) and the presence of some previous risk factor (obesity, previous obstetric or chronic pathology) with two possible answers (yes/no) are considered. Fear of childbirth was measured by its influence on the choices about childbirth seen in other works [27–29]. It was measured using the W-DEQ-A questionnaire validated in Spanish [30]. It is a self-administered questionnaire with 33 items, each of them evaluating a feeling on a numerical scale from least (0) to most (5). In items 2, 3, 6, 7, 8, 11, 12, 15, 19, 20, 24, 25, 27 and 31, the order of the scores is reversed. The minimum score of the questionnaire is 0 and the maximum 165.

### Statistical analysis

For each of the statements presented, descriptive statistics were used to analyse quantitative data, and the response percentages were calculated. For the items of greater variability (< 75% of the sample “agree” or “totally agree”), regression models were built to test the effect of sociodemographic variables and fear of childbirth. Each definitive model was built following a backward strategy using likelihood ratio tests as selection criteria (*p*-value < 0.05).

Analyses were carried out with SAS, version 9.4 (Cary, North Carolina, USA).

## Results

Two hundred forty seven women gave their consent and answered the questionnaires between weeks 8 and 41 of gestation. The descriptive characteristics of the sample are presented in Table 1.

**Table 1:** Descriptive characteristics of participating women

		Total n	%
Age			
	< 30	40	16,2
	30–34	105	42,5
	35–39	74	30
	≥40	28	11,3
Parity			
	None	173	70,3
	One or more	73	29,7
Nationality			
	Spanish	216	87,4
	Foreign	31	12,6
Level of education			
	Low (Primary)	25	10,2
	Medium(Secondary)	86	35,2
	High (University)	133	54,5
PaidWork			
	Yes	176	73,9
	No	62	26,1
RiskFactors			
	Yes	138	55,9
	No	109	44,1
W-DEQ-A			
Mean (SD)		54,82	20,18

**Fig. 4:**
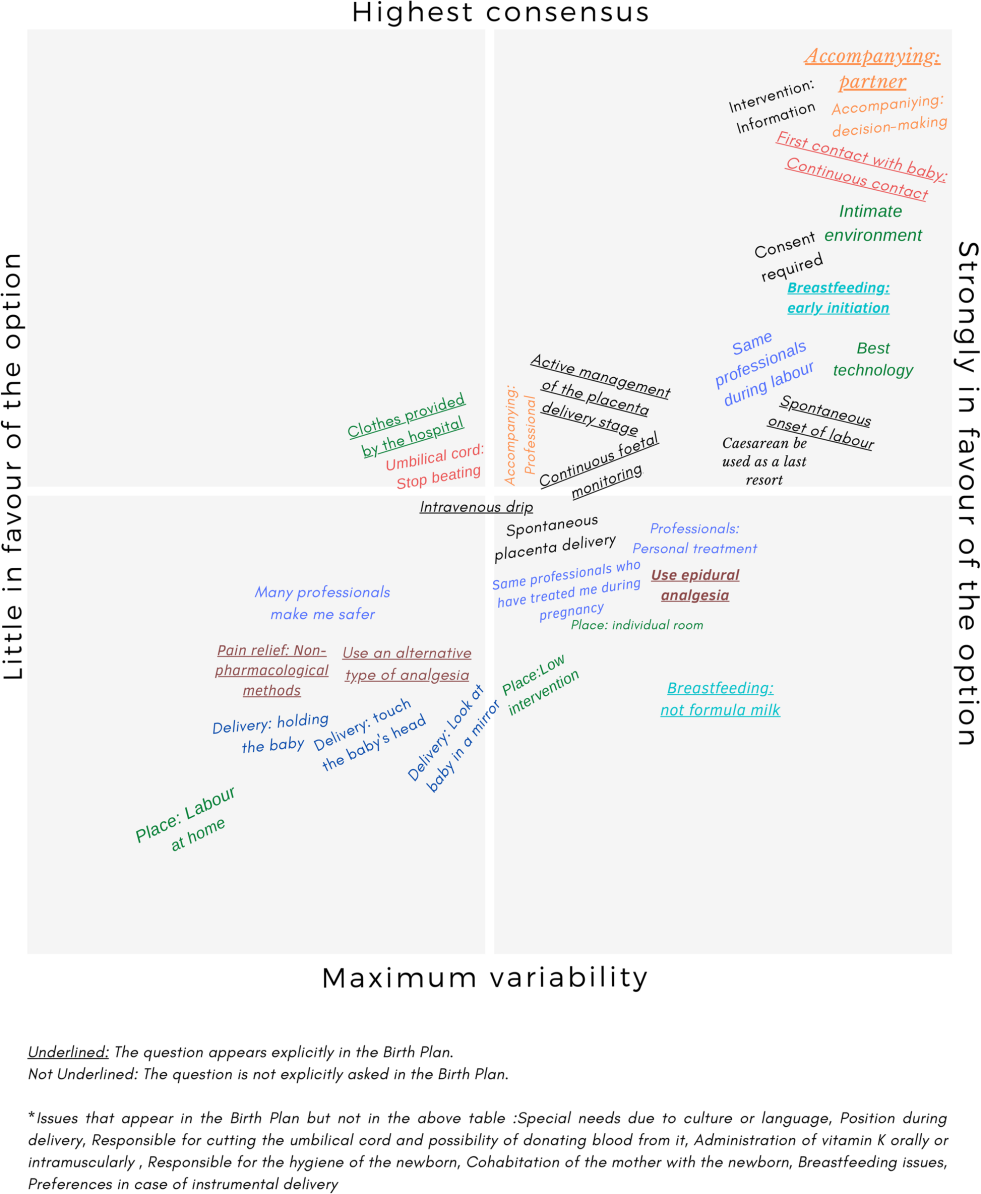
Figure illustrating the degree of agreement with the options/questions and variability, indicating which of them are included in the reference delivery plan

The percentage of women who expressed their agreement, neutrality or disagreement with each of the statements referring to childbirth and immediate postpartum is presented in Figs. 2 and 3. Most women consider it very important to be accompanied by their partner during the birth, as well as for the centre to offer high technology and for the atmosphere to be as intimate as possible. They also attach great importance to the first contact with the baby, which must be continuous, and to being informed and asked for their consent before any intervention is carried out. More technical issues, such as the cutting of the umbilical cord or that there are many professionals attending to them, are not issues that they prioritise. Figure 4 is a graphical representation of what issues are most relevant to women and whether they are taken into consideration in the Ministry’s childbirth plan. There are items that are underlined: those are included in the Ministry’s Childbirth Plan. On the other hand, the items at the top-right are considered very important by the majority of women, and the items at the top-left are issues to which women mostly attach less importance. The items at the bottom are items with more variability. We can see that there are some almost unanimous preferences for women, such as the need for information, consent before interventions, or the type of environment they want for childbirth that are not taken into consideration in the Birth Plan. Others, like accompanying partner or continuous contact with the baby are very relevant and figure inside the Birth Plan.

Table 2 shows the adjusted models of the relationship between agreement with the options with the greatest variability (less than 75% “agree” or “strongly agree”) and sociodemographic variables, parity, risk factors and fear of childbirth. Thus, having more fear of childbirth is related to the request for more professional attention and a lower need for close interaction with the newborn during childbirth. Having a previous child, however, is associated with a greater preference for this early contact with the newborn, wanting to see it, touch it, and even extract it during delivery. The educational level seems to be associated with the preference for a delivery with low professional intervention. Having risk factors or previous pathologies is related to a greater preference for health care, while maintaining an active participation in delivery. Finally, nationality is associated with less preference for epidural anesthesia, but more interest in sustained professional care.

**Table 2:** Adjusted models of the relationship between agreement with the options with the greatest variability and the sociodemographic variables, parity, risk factors and fear of childbirth. Significant associations (*p* ≤ 0,05)

	*Fear of childbirth	Age	Multiparous	School educa-tion	Higher educa-tion	No paid job	Risk fac-tors	Foreign mother
Environment								
*It is very important for me to have a single room*				−0.43	−0.62			
*I value the fact that medical intervention in the center should only be offered when absolutely essential. My priority is a natural delivery whenever possible.*	−0.01							
*I would like to wear the clothes provided by the hospital*				0.05	−0.17			0.45
Professionals								
*It is important for me that the professionals who are going to attend me introduce themselves and inform me about their training/functions.*			0.22					
*It is important to be accompanied by the same professionals who have treated me during pregnancy (gynecologist, midwife)*	0.01						0.2	
*The more professionals that attend me (gynecologists, midwives or pediatricians), the safer I will feel.*	0.01						0.35	0.53
BirthPartner								
*It is important that a health professional or doula should accompany me, as long as possible, during the dilation period*	0.01							0.31
Pain relief								
*I would like to use epidural analgesia*		0.03		0.18	−0.18			−0.39
*I would like to use a type of analgesia that I know about (such as hot water baths, local heat, nitrous oxide, lumbar massage, sterile water injections or tens)*		−0.03		0.53	0.64		0.21	
*I would prefer to use non-pharmacological methods to relieve pain during labor*						−0.35		0.33
Care and intervention during childbirth								
*Although it may limit my movement, I prefer continuous fetal monitoring because I think it is safer for the baby*			0.35	−0.2	− 0.45		0.21	
*I prefer active management of the placenta delivery stage, with the help of a drug, if this reduces the possibility of postpartum hemorrhage*			−0.29	− 0.31	− 0.05			
*I prefer a spontaneous placenta delivery, without any intervention*						−0.27		
*Being put on an intravenous drip gives me security and does not detract from comfort*								
Delivery								
*I would like to be able to see the baby in a mirror as it comes out*	−0.01		0.27				0.22	
*I would like to be able to touch the baby’s head when it crowns*	− 0.01		0.29				0.34	
*I would like to be able to catch the baby*	−0.01		0.28				0.43	−0.41
First contact with the newborn								
*It is important for me to wait for the umbilical cord to stop beating before clamping it.*			0.2	0.4	0.42			

## Discussion

Among the options that are usually part of birth plans in our area, there are some that, in addition to being considered important by the women, generate a high degree of consensus. Those are the options that reflect the most emotional and relational aspects, the human part of childbirth. They include the possibility of deciding on the accompanying person, early and continuous contact with the baby, or favouring early initiation of breastfeeding. The results coincide with those obtained by Barnes et al. in 2022 with women who were facing a scheduled caesarean section. The authors found that more than 90% requested immediate skin-to-skin contact, the participation of their support person, and help with the initiation of breastfeeding [31]. This need to maintain a sense of control and be surrounded by the people closest to the woman is the most frequent finding in the literature, both in home and hospital births [13, 15, 17, 32–34] and reflects what Westergren calls “dependent autonomy” [5, 35]. It seems evident, therefore, that this care should constitute the basis of childbirth care, rather than being an option suggesting the possibility of choosing other care. The same would happen with the need for information and the request for consent regarding the interventions to be performed, or being in a private space with access to technology in the event of an emergency, which were valued as very important. It would not make sense either that they should be optional.

Other issues in the birth plan, however, show greater variability, which would justify their use for providing different care to the woman according to her preferences. These are the options related to medical interventions during dilation (monitoring, infusion) or placenta delivery (managed or spontaneous); the type of analgesia and participation in delivery are also included. More than a third of the women had a neutral opinion on these clinical questions, a result which was like that found by Barnes et al., when asked about matters such as umbilical cord clamping [31]. This lack of position may be related to a lack of information about the advantages and disadvantages of these techniques [36]. It would be necessary, before making any decision, for women to have exhaustive and unbiased information, knowing some risks or consequences of certain decisions. For example, it has been observed that uterine atony is responsible for 41.2% of peripartum hysterectomies [37] an intervention that can have dramatic consequences even for the life of the mother. Precisely the indication of active management of placenta delivery aims to prevent this atony. An informed decision needs to be aware of these risks.

It is also possible that the apparent lack of interest in this type of action is because they reflect matters that are of interest to the health care professionals rather than the women [4, 12, 38, 39]. Or that, given the unpredictability of the birth process, they prefer to decide some issues only when the time comes, for example, for fear of facing up to the various scenarios. Following the same approach, it would also be justified to attend to the preferences of women in terms of the intensity and mode of participation or presence of the professionals during all stages of the delivery process. These questions are of moderate variability and should also be considered: 1) because they will inevitably influence critical issues for them, such as the desire for an intimate environment, and 2) because this variability is related to other variables, such as fear of childbirth, which we will discuss further on.

Finally, there are issues that are not included in the birth plan because they are not optional at the moment. For example, in our country, home birth is not financed by the public health system and must be paid for in full, so it is not usually included as an option. In our sample, despite the fact that the question referring to home delivery had a high percentage of disagreement, 10% of the women agreed with this option. Nevertheless, only 0.32% of births in Spain occur at home [40]. The difference between considering an alternative and carrying it out may be due to the perception of an ideal home birth as a natural and intimate event, but ultimately women do not want to assume the possible associated risk [12, 15]. In addition, in this public health service, caesarean section or induction cannot be requested by the woman, and are performed only under medical criteria. As seen in other studies, [32, 41], for more than 80% of the women in our sample, induction of labour or caesarean section would be the last option. It is possible that both circumstances go hand in hand, since the reasons why women choose one type of delivery or another include encouragement or dissuasion by health professionals, cultural influence, or access to information [32].

Agreement or disagreement with some options is associated with certain factors. Women who already have a previous child show greater agreement with the options in which more contact with the baby is offered, such as seeing it, touching it, or even helping with the delivery. The prior existence of a bond with other children seems to facilitate the creation of the new bond and the search for greater contact [15, 42, 43]. Women from other countries in our study were more favourable to home birth, non-pharmacological pain relief and the immediate placement of the baby at the breast, but they also requested more support and professional intervention. Cultural differences regarding childbirth expectations have frequently been seen [29]. Foreign women could find childbirth much more medicalised than in their places of origin and do not consider it necessary, but do not reject the resources available [14, 15].However, women with some risk factor such as a previous chronic disease, a history of prematurity or previous foetal death, consumption of toxic substances or pathologies in the current pregnancy show a need for greater care with more professional presence and foetal control on the one hand, and on the other a greater desire for contact and relationship from the moment of delivery. The existence of a risk pregnancy is an intense experience for both the woman and the family, and frequently involves anxiety and fear [44], which would be associated with a greater need for medicalisation of the birth, which perhaps the woman herself tries to humanise.

Other variables that are associated with a greater or lesser acceptance of medical interventions during childbirth, however, could be modified. Women with a higher educational level, preferred non-pharmacological methods of pain relief, late clamping of the umbilical cord, or intermittent monitoring, as they may have more information about current issues and good practice. Women with higher scores on fear of childbirth, however, agree more with medical interventions during childbirth, continuous professional presence, and agree less with participation in delivery. This result coincides with previous studies [18, 27, 41] in which it is shown that both fear and greater medical intervention in childbirth lead to higher morbidity rates and worse postpartum recovery.

### Limitations

This study has some limitations. The use, in part, of snowball sampling, added to that carried out in the midwives’ offices, may have resulted in the presence of women with more personal resources; although measures have been taken to reduce selection bias: the recruitment was carried out by 25 primary-care midwives located in both rural and urban centres of population, with different socioeconomic characteristics. It is true that the participation of immigrant women was low compared to the volume of deliveries they currently represent (28% [23]), probably due to the language requirement. This low participation coupled with the variety of countries of origin does not allow for comparison of cultural practices.

Other methodological limitation of this study is that it was not originally designed to extrapolate the data to other populations, but is part of another investigation whose objective was “to create a tool to measure the needs of women during pregnancy” [22]. This means that the sample had to be representative of our specific population, so the results may not be generalizable to other populations of pregnant women with different characteristics.

The exploratory and descriptive nature of the study does not allow conclusions to be drawn about causality between the characteristics of the women and the preferences expressed, in addition to the possibility that these preferences may vary over time and, above all, at the time of birth. Further research with longitudinal designs would be useful to establish the temporal or causal relationship and the extent to which the experience of pain modifies these preferences.

In all likelihood, the results shown in this study will be similar to those that can be found in other Western countries, but it is more unlikely that the study can be extrapolated to other populations with different resources and cultures about childbirth.

Shared decision making and birth plan are a relevant issue in pregnancy and childbirth care. In this context we introduce a reflection on the usefulness of certain questions in the birth plan. It is clear that some questions have to be part of routine care, those for which there is a high degree of unanimity among women. Attention should be focused on the questions that generate the greatest variability in the answers. These tend to be the more technical questions, the advantages or disadvantages of which women are unaware and about which it is useful for them to think and make decisions.

## Conclusions

The birth plan currently offered is not fully adapted to women’s areas of interest. To support the woman in making shared decisions about childbirth and the arrival of the newborn, it is important to know what is really relevant to her. The findings suggest that having safety, maintaining family contact and a high degree of control and involvement in decision-making are valued by the vast majority of women. Consequently, they should be essential in all maternity services as the basis of childbirth care. The clear majority position on the most emotional issues, such as skin-to-skin contact, breastfeeding or partner support, contrasted with their lack of interest or agree in choices more closely related to clinical practice such as the type of delivery, the moment of clamping the umbilical cord or the attitude or posture in the expulsion stage. However, most of the time, the birth plan places a great deal of emphasis on these technical issues.

The completion of this birth plan during pregnancy could be considered a declaration of intent, but it should be adjusted later in the specific situations of childbirth [33, 45]. Asking the right questions, only the necessary ones, and providing the information to make reflection possible, will undoubtedly result in more satisfactory birth experiences and a reduction in unnecessary medical interventions.

### Supplementary Information

**Additional file 1.**

## Data Availability

All data generated or analysed during this study are included in this published article [and its supplementary information files].
